# Vaccine-Induced Immune Thrombotic Thrombocytopenia (VITT): An Immunopathogenic Model of Dysregulated Vaccine-Triggered Immunity

**DOI:** 10.3390/vaccines14030225

**Published:** 2026-02-28

**Authors:** Carmine Siniscalchi, Manuela Basaglia, Antonella Tufano, Egidio Imbalzano, Pierpaolo Di Micco

**Affiliations:** 1 Department of Internal Medicine, Parma University Hospital, 43100 Parma, Italy; csiniscalchi84@gmail.com (C.S.); mbasaglia80@gmail.com (M.B.); 2 Department of Internal Medicine, “Federico II University” Napoli University Hospital, 80078 Naples, Italy; atufano@unina.it; 3 Department of Internal Medicine, Messina University Hospital, 98121 Messina, Italy; egidio.imbalzano@unime.it; 4 UOC Medicina Interna, P.O. Pozzuoli, ASL Napoli2 nord, 80078 Pozzuoli, Italy

**Keywords:** vaccine, thrombocytopenia, thrombosis, immune

## Abstract

Background/Objectives: Vaccine-induced immune thrombotic thrombocytopenia (VITT) is a rare but severe immune-mediated adverse event associated with adenoviral vector-based SARS-CoV-2 vaccines. Beyond its clinical relevance, VITT provides a unique human model of vaccine-triggered autoimmunity and immune-thrombosis. This review critically reassesses the immunopathogenic framework of VITT in light of recent evidence. Methods: We conducted a structured narrative review of studies published between 2021 and 2025, focusing on clinical, epidemiological, and mechanistic data relevant to PF4 immunogenicity, platelet activation, and long-term outcomes. Results: Current evidence supports a multistep model in which adenoviral vector components form immunogenic PF4–polyanion complexes that induce high-affinity anti-PF4 IgG antibodies. These antibodies activate platelets via FcγRIIa, amplify complement signaling, promote neutrophil extracellular trap formation, and drive endothelial perturbation, establishing a self-sustaining thrombo-inflammatory loop. Recent longitudinal studies refine earlier interpretations by distinguishing persistent anti-PF4 seropositivity from sustained platelet-activating capacity. Epidemiological data support platform-enriched risk rather than absolute platform exclusivity, with a proposed mechanistic “border zone” for incomplete phenotypes. Conclusions: VITT represents a tractable human model of vaccine-induced autoimmunity in which innate immune activation and multivalent antigen presentation converge to break tolerance. Updated evidence clarifies antibody persistence, platform enrichment, and translational implications, while highlighting unresolved questions regarding host susceptibility and long-term immune regulation.

## 1. Introduction

Vaccine-induced immune thrombotic thrombocytopenia (VITT) is a newly described, immune-mediated thrombo-inflammatory disorder that was first recognized in early 2021, shortly after the global rollout of adenoviral vector-based SARS-CoV-2 vaccines, including ChAdOx1 nCoV-19 (AstraZeneca) and Ad26.COV2.S (Johnson & Johnson/Janssen) [1,2,3].

Vaccination remains one of the most effective public health interventions for the prevention of infectious diseases, with an unparalleled impact on morbidity and mortality worldwide. The rapid development and deployment of SARS-CoV-2 vaccines during the COVID-19 pandemic represented an unprecedented scientific and regulatory achievement, enabling large-scale immunization within an extremely compressed timeframe. Alongside these benefits, the global vaccination campaign also highlighted the importance of robust safety surveillance systems capable of detecting, characterizing, and contextualizing rare adverse events following immunization.

Although serious immune-mediated adverse events are exceedingly uncommon, their occurrence can have a disproportionate impact on public perception, vaccine confidence, and policy decisions. For this reason, understanding the biological mechanisms underlying rare vaccine-associated syndromes is not only a clinical or immunological challenge but also a critical component of evidence-based vaccinology and regulatory science. Mechanistic insight allows distinction between coincidental events and true vaccine-triggered immune phenomena, supports rational risk stratification, and informs the design of safer next-generation vaccine platforms.

The syndrome was initially referred to as vaccine-induced prothrombotic immune thrombocytopenia (VIPIT) in early reports, reflecting uncertainty regarding its relationship with thrombosis. As the clinical and immunological features became clearer, the term vaccine-induced immune thrombotic thrombocytopenia (VITT) was adopted to emphasize the central role of immune-mediated thrombosis. In this review, we use the term VITT throughout for consistency with current consensus.

The syndrome is characterized by the paradoxical coexistence of severe thrombocytopenia and thrombosis, frequently affecting unusual vascular beds such as the cerebral venous sinuses, splanchnic veins, and, in some cases, arterial territories [1,2]. Laboratory findings typically include markedly elevated D-dimer levels, hypofibrinogenemia, and the presence of high-titer IgG antibodies directed against platelet factor 4 (PF4) in the absence of prior exposure to heparin [3,4,5].

The recognition of VITT represented a pivotal moment in vaccinology and immuno-thrombosis research. Although rare, its severity and distinctive immunopathological features rapidly attracted international attention, leading to the identification of a novel form of autoimmune, antibody-mediated thrombosis that shares mechanistic similarities with heparin-induced thrombocytopenia (HIT) yet differs in trigger, antibody profile, and clinical expression [6,7,8]. Importantly, VITT does not represent a nonspecific inflammatory coagulopathy but rather a highly specific immune response, in which the adaptive immune system generates pathogenic antibodies against a self-protein following exposure to a vaccine-derived trigger [7,9].

From a broader perspective, VITT provides a unique human model of dysregulated vaccine-triggered immunity. Vaccines are designed to stimulate robust innate and adaptive immune responses to confer protection against infectious pathogens. However, in exceedingly rare circumstances, this immune activation may deviate toward pathological autoimmunity, particularly when self-antigens form complexes with strongly immunogenic exogenous molecules [6,10]. In VITT, PF4 (a small, positively charged chemokine released by activated platelets) binds to negatively charged vaccine-derived components, including adenoviral proteins, free DNA, and other polyanionic constituents, forming multimolecular complexes that expose neoepitopes capable of breaking immune tolerance [11,12,13].

The resulting anti-PF4 IgG antibodies engage FcγRIIa receptors on platelets, triggering massive platelet activation, aggregation, and the release of procoagulant extracellular vesicles, thereby initiating a feed-forward thrombo-inflammatory loop [3,5,14]. Concomitantly, immune complexes activate the complement cascade, stimulate neutrophil extracellular trap (NETs) formation, and induce endothelial perturbation, further amplifying thrombosis and vascular injury [15,16,17,18]. This integrated model of immune-mediated thrombosis provides a mechanistic explanation for the rapid clinical deterioration and extensive thrombotic burden observed in affected patients.

Beyond its immediate clinical implications, the study of VITT has reshaped our understanding of immune-mediated coagulopathies and has highlighted the delicate balance between protective immunity and pathological autoimmunity. Lessons learned from this syndrome extend far beyond COVID-19 vaccines, offering valuable insights into the design of next-generation vaccine platforms, the identification of immune-mediated adverse events, and the development of predictive biomarkers for immune dysregulation [10,19].

In contrast to earlier reviews published between 2021 and 2023, which primarily focused on defining the clinical phenotype and establishing the PF4/FcγRIIa-centered cascade, the present 2025 reassessment addresses whether emerging data materially modify prior assumptions. Specifically, recent longitudinal and mechanistic evidence challenges three earlier simplifications: (a) the implicit equation between anti-PF4 ELISA positivity and sustained pathogenicity; (b) the early framing of adenoviral vectors as qualitatively exclusive triggers rather than quantitatively enriched platforms; and (c) the assumption that antibody generation represents a static autoimmune event rather than a dynamic and potentially reversible immune process. By explicitly distinguishing binding from functional antibodies, probabilistic enrichment from exclusivity, and acute immune activation from long-term immune remodeling, this review proposes a refined conceptual model in which VITT is understood not as a fixed entity but as a spectrum phenomenon embedded within a broader immunological context.

In this broader context, rare immune-mediated thrombotic syndromes assume particular relevance, as they provide a window into the mechanisms through which protective immune activation may, in exceptional circumstances, deviate toward pathological autoimmunity. Unlike nonspecific inflammatory adverse events, these syndromes are characterized by reproducible immunological signatures, identifiable autoantibodies, and defined effector pathways. As such, they offer a unique opportunity to dissect causal links between vaccine components, innate immune sensing, adaptive immune responses, and clinical phenotype.

In the present review, we synthesize and critically appraise clinical, immunological, and experimental evidence to delineate the molecular and cellular mechanisms underlying VITT and to define key uncertainties that should guide future research and translational strategies. We discuss its relationship with HIT and COVID-19-associated coagulopathy, explore why this syndrome appears platform-enriched in association with adenoviral vector vaccines, and examine the broader implications of VITT as a model of vaccine-induced autoimmunity and immune-mediated thrombosis.

## 2. Methods (Literature Search and Selection)

This article is a narrative, non-systematic review. To improve transparency and ensure adequate coverage of the rapidly evolving VITT literature, we conducted a structured literature search focused on clinical epidemiology, immunopathogenesis, and management.

The final search was performed on 15 December 2025. The core PubMed search string was: (“vaccine-induced immune thrombotic thrombocytopenia” OR VITT OR VIPIT OR “thrombosis with thrombocytopenia syndrome”) AND (“platelet factor 4” OR PF4 OR “anti-PF4” OR FcγRIIa OR “adenoviral vector vaccine”). This search yielded approximately 200 records. After full-text assessment, 40 core studies were included for detailed synthesis; however, additional cohort reports, pharmacovigilance updates, and mechanistic studies published between 2023 and 2025 were incorporated during manuscript preparation to ensure contemporaneous coverage. Particular emphasis was placed on longitudinal antibody behavior, post-policy epidemiology, comparative immunology, and translational laboratory investigations published after 2023, as these domains are central to reassessing prior assumptions rather than reiterating early descriptive reports. We prioritised peer-reviewed studies published between 2021 and 2025, including observational cohorts, pharmacovigilance analyses, mechanistic/experimental studies, and consensus guidance documents, with particular emphasis on data published from 2023 to 2025 to support an updated synthesis. Preprints were considered only when highly relevant to emerging topics (e.g., anti-PF4 persistence) and clearly labelled as such. Reference lists of key articles were screened to identify additional relevant sources (“snowballing”).

To minimise selective citation, we ensured representation of major post-2023 cohort follow-up studies, platform-specific pharmacovigilance updates, and mechanistic investigations across independent groups.

We included articles that provided (a) clinical case definition/diagnostic frameworks, (b) epidemiological estimates and outcome data, (c) mechanistic insights into PF4–polyanion complex formation and anti-PF4 antibody generation, and (d) therapeutic approaches and follow-up considerations. Articles were excluded when they did not address VITT or when they focused exclusively on nonspecific post-vaccination thrombotic events without anti-PF4 evidence. The synthesis was qualitative and aimed to combine established mechanisms with critical discussion of unresolved questions and translational implications.

## 3. Epidemiology and Clinical Phenotype of VITT

Vaccine-induced immune thrombotic thrombocytopenia (VITT) was first recognized in early 2021 through clusters of unusual thrombotic events temporally associated with adenoviral vector-based SARS-CoV-2 vaccination [1,2,20,21]. These initial reports rapidly led to international surveillance efforts, which confirmed the existence of a distinct clinical syndrome characterized by thrombosis in atypical anatomical sites, severe thrombocytopenia, and high-titer anti-PF4 antibodies in individuals without prior heparin exposure [3,4,5,21]. Although the overall incidence of VITT is low, estimated at approximately 1–2 cases per 100,000 vaccine recipients, the syndrome is associated with substantial morbidity and mortality, particularly when diagnosis and treatment are delayed [12,13,22,23].

More recent pharmacovigilance analyses up to 2023 confirm the rarity of vaccine-induced TTS/VITT, while highlighting substantial geographic variability linked to vaccine deployment strategies and evolving risk-mitigation policies [22].

Reported incidence rates of VITT have shown relevant geographic variability, influenced not only by biological factors but also by vaccine deployment strategies, surveillance intensity, and diagnostic awareness. The syndrome was most frequently identified in countries where adenoviral vector vaccines were widely used in younger populations during the early phases of the COVID-19 vaccination campaign, particularly in several European settings. In contrast, regions that prioritized mRNA-based platforms or limited adenoviral vector vaccines to older age groups reported markedly fewer cases.

Importantly, this heterogeneity underscores the strong interaction between epidemiology and public health policy. Following regulatory restrictions, age-based recommendations, or withdrawal of specific vaccine products, the incidence of newly diagnosed VITT cases declined substantially. These observations support a causal association while also illustrating how population-level risk can be dynamically modulated by vaccination strategies, an aspect that is highly relevant for regulatory decision-making and future vaccine deployment.

Epidemiological studies have consistently shown that VITT predominantly affects younger adults, often under 50 years of age, with a slight female predominance in early reports [11,12,13]. However, subsequent analyses suggest that this apparent sex difference may partly reflect vaccination policies and demographic factors rather than a true biological predisposition [14]. Importantly, no clear association with traditional thrombotic risk factors has been identified, underscoring the immune-mediated nature of the disease [1,3].

Clinically, VITT typically presents between 5 and 30 days following vaccination, with a median onset around 10–14 days [1,2,11]. Patients frequently report severe headache, visual disturbances, abdominal pain, nausea, dyspnea, or focal neurological deficits, reflecting thrombosis in the cerebral venous sinuses, splanchnic veins, pulmonary arteries, or, less commonly, arterial territories [2,11]. Cerebral venous sinus thrombosis (CVST) and splanchnic vein thrombosis are particularly characteristic of VITT and are rarely observed together with thrombocytopenia in other clinical contexts, making their association a hallmark of the syndrome [1,4]. Notably, increasing recognition of prodromal or atypical presentations has expanded the clinical spectrum of VITT, reinforcing the importance of maintaining a high index of suspicion in the appropriate temporal and laboratory context.

Laboratory findings are highly distinctive. Profound thrombocytopenia is a consistent feature, often with platelet counts below 50 × 10^3^/μL, accompanied by extremely elevated D-dimer levels, frequently exceeding 20,000 ng/mL, and low fibrinogen concentrations, reflecting ongoing consumptive coagulopathy. A defining diagnostic criterion is the presence of high-titer IgG antibodies against PF4, detected by enzyme-linked immunosorbent assay (ELISA), which strongly activate platelets in functional assays despite the absence of heparin [3,4,18].

Early in the pandemic, VITT was associated with mortality rates exceeding 40%, particularly in patients with CVST complicated by intracranial hemorrhage or massive splanchnic thrombosis [1,11]. However, the rapid dissemination of diagnostic algorithms and treatment guidelines, including early administration of high-dose intravenous immunoglobulin (IVIG) and non-heparin anticoagulation, has led to a marked improvement in outcomes, with recent cohorts reporting mortality rates below 10–15% [14,24].

### Long-Term Evolution and Post-Policy Landscape (2023–2025)

As vaccine programs evolved and adenoviral vector use declined or became platform-enriched association in many settings, the clinical landscape of VITT also changed. More recent real-world safety studies and pharmacovigilance analyses suggest fewer incident cases in jurisdictions where adenoviral vaccines were de-emphasized, while improved clinical awareness and standardized diagnostic pathways likely contributed to better outcomes in diagnosed patients. These developments reinforce that clinical impact depends not only on biological mechanisms but also on vaccine policy, case ascertainment, and time-to-treatment.

Large-scale linked-health-record cohort evidence further supports a platform-enriched (rather than platform-exclusive) pattern of rare harms, including vaccine-induced thrombotic thrombocytopenia after first ChAdOx1 vaccination, within a broader landscape of generally reduced thrombotic event rates after vaccination overall [25].

A key unresolved issue that has gained attention in recent years is the long-term behavior of anti-PF4 antibodies after acute VITT. Emerging data indicate that anti-PF4 seropositivity may persist for months, with serial quantitative testing and functional assays documenting prolonged ELISA positivity in a subset of patients [26]. This uncertainty has direct clinical implications for follow-up strategies, duration of anticoagulation, and decisions regarding re-vaccination or exposure to potential PF4-binding triggers.

Importantly, recent cohort studies have clarified that serological persistence of anti-PF4 IgG does not necessarily equate to sustained pathogenic potential. In several longitudinal analyses, ELISA positivity persisted for months or even years in a subset of patients, whereas functional platelet-activation assays (including serotonin release assays, PF4-enhanced platelet activation tests, and FcγRIIa-dependent flow-based assays) became negative over time in most patients. This ELISA–function dissociation has been specifically documented in longitudinal follow-up cohorts using PF4-enhanced platelet activation platforms (e.g., HIPA/PIPA-type approaches) alongside binding assays [26]. This distinction is clinically relevant: while ELISA detects binding antibodies, only a fraction retain the ability to trigger FcγRIIa-mediated platelet activation. Thus, persistence of seropositivity should not automatically be interpreted as ongoing thrombotic risk. This refinement significantly nuances earlier interpretations, which tended to conflate antibody persistence with sustained pathogenicity, and supports a more individualized and function-oriented follow-up strategy.

Taken together, these 2023–2025 developments support the need for an updated synthesis that extends beyond acute presentation to incorporate longer-term follow-up, evolving epidemiology, and persisting mechanistic controversies.

Importantly, VITT must be distinguished from other thrombotic or thrombocytopenic conditions occurring after vaccination, such as incidental venous thromboembolism, immune thrombocytopenia, or COVID-19-associated coagulopathy [27,28]. The combination of thrombocytopenia, markedly elevated D-dimer levels, and anti-PF4 antibodies defines a unique immunopathological entity that is mechanistically and clinically distinct from these disorders [7,9].

Taken together, the epidemiological and clinical features of VITT highlight a rare but highly specific immune-mediated thrombotic syndrome. Its consistent temporal association with adenoviral vector vaccines, stereotypical laboratory profile, and dramatic clinical course strongly support a causal immunopathogenic mechanism rather than a coincidental adverse event [6,10]. Key clinical and laboratory hallmarks of VITT are summarized in Table 1.

## 4. Immunopathogenesis of VITT

Immune-mediated thrombosis represents a paradigm in which inflammatory and adaptive immune mechanisms directly interface with hemostatic pathways. Platelets, traditionally viewed as passive effectors of coagulation, are now recognized as active immune cells capable of sensing danger signals, interacting with leukocytes, and amplifying inflammatory responses. In this context, immune complexes, Fc receptor engagement, complement activation, and neutrophil extracellular trap formation constitute shared mechanistic axes across several immunothrombotic disorders.

VITT exemplifies an extreme and highly specific form of this interface, in which a discrete autoantibody response is sufficient to trigger a fulminant thrombo-inflammatory cascade. Understanding its immunopathogenesis therefore requires integration of platelet biology, innate immune sensing, adaptive immune tolerance, and effector cell activation within a unified framework.

The defining hallmark of vaccine-induced immune thrombotic thrombocytopenia (VITT) is the generation of pathogenic IgG antibodies directed against platelet factor 4 (PF4), which drive a fulminant thrombo-inflammatory cascade through platelet, innate immune, and endothelial activation [3,4,5,6,7]. The immunopathogenesis of VITT can be conceptualized as a multistep process involving: formation of immunogenic PF4–polyanion complexes; breaking of immune tolerance and clonal expansion of anti-PF4 B cells; and Fc-mediated cellular activation leading to immune-thrombosis.

### 4.1. Formation of Immunogenic PF4-Polyanion Complexes

PF4 is a small, positively charged CXC chemokine stored in platelet α-granules and rapidly released upon platelet activation. Owing to its strong cationic properties, PF4 has a high affinity for negatively charged molecules, including glycosaminoglycans, bacterial surfaces, nucleic acids, and heparin [15,16]. In VITT, components of adenoviral vector vaccines act as functional polyanions capable of binding PF4 and inducing the formation of large, multimolecular complexes [6,11,12].

Experimental studies have demonstrated that adenoviral capsid proteins, residual free DNA, and other vaccine-associated polyanionic constituents readily associate with PF4, generating stable complexes with enhanced immunogenicity [11,12,13]. These complexes expose cryptic neoepitopes on PF4 that are not accessible in its native conformation, thereby rendering PF4 a “danger-associated self-antigen” [6,10]. Importantly, PF4–polyanion interactions are not intrinsically pathological and occur physiologically in multiple inflammatory settings, including bacterial infections and tissue injury. What distinguishes VITT is not merely complex formation but the acquisition of sufficient immunogenicity to drive a sustained adaptive immune response. Factors influencing this transition remain incompletely defined and likely include the size, stability, and multivalency of PF4-containing complexes, as well as the inflammatory context in which they are encountered.

Moreover, experimental evidence suggests that only specific configurations of PF4–polyanion complexes expose the neoepitopes recognized by pathogenic antibodies. This observation supports a qualitative, rather than purely quantitative, model of antigenicity and helps explain why widespread exposure to PF4-binding polyanions does not routinely result in autoimmunity.

This mechanism closely resembles the pathogenesis of autoimmune HIT, in which PF4–heparin complexes trigger a similar loss of immune tolerance [8,9].

### 4.2. Breaking of Immune Tolerance and Anti-PF4 Antibody Generation

The formation of PF4-polyanion complexes provides the first signal for immune activation; however, the generation of high-affinity, class-switched IgG antibodies indicates the involvement of T-cell-dependent adaptive immune responses [17,19]. High-throughput sequencing of B-cell receptors in patients with VITT has revealed oligoclonal expansions with extensive somatic hypermutation, consistent with antigen-driven clonal selection and affinity maturation [19]. More recently, mechanistic human and experimental data have identified adenoviral protein pVII as a plausible inciting antigen and have directly linked somatic hypermutation to the antigenic ‘shift’ from adenoviral components toward PF4, providing a concrete mechanistic layer beyond earlier descriptive models [29].

Innate immune sensing pathways play a crucial role in this process. Adenoviral DNA and associated pathogen-associated molecular patterns (PAMPs) activate Toll-like receptor 9 (TLR9) and cytosolic DNA sensors, such as the cGAS–STING pathway, in antigen-presenting cells. This leads to robust type I interferon and pro-inflammatory cytokine production, creating an immunological milieu that favors the activation of autoreactive B cells recognizing PF4-containing complexes [6,10]. In this context, the vaccine acts not merely as an antigenic stimulus but as a potent adjuvant capable of lowering the threshold for autoantibody generation.

From an immunological perspective, the emergence of high-affinity, class-switched anti-PF4 IgG antibodies implies a failure of peripheral tolerance rather than a nonspecific polyclonal response. In healthy individuals, autoreactive B cells recognizing PF4 are thought to be either deleted, rendered anergic, or actively regulated. In VITT, however, the convergence of strong innate immune activation and multivalent antigen presentation appears sufficient to override these checkpoints.

Recent molecular analyses have demonstrated oligoclonal expansion of anti-PF4 B cells with extensive somatic hypermutation, consistent with antigen-driven selection rather than bystander activation. This finding aligns VITT with established autoimmune paradigms and supports its conceptualization as a vaccine-triggered autoimmune process rather than an idiosyncratic toxic effect. Nevertheless, the host-specific factors that permit this breach of tolerance, such as immunogenetic background, prior immune priming, or transient alterations in regulatory networks, remain largely undefined.

### 4.3. VITT in the Spectrum of Vaccine-Associated Autoimmunity

The concept of VITT as a model of vaccine-induced autoimmunity gains additional strength when placed in the broader context of post-vaccination autoimmune phenomena. Rare immune-mediated conditions such as immune thrombocytopenia, Guillain–Barré syndrome, myocarditis, and narcolepsy have been described after different vaccines, suggesting that, in susceptible individuals, immune activation may occasionally breach tolerance mechanisms.

In VITT, several features argue for a bona fide autoimmune process rather than nonspecific inflammation. These include the high specificity of anti-PF4 antibodies, their class-switched IgG profile, and evidence of antigen-driven B-cell clonal expansion with somatic hypermutation. Such findings are consistent with a T-cell-dependent response occurring in an inflammatory milieu capable of transiently disrupting peripheral tolerance checkpoints.

Adenoviral vector vaccines provide strong innate immune stimulation through DNA sensing pathways (e.g., TLR9 and cGAS–STING), generating a cytokine environment that may lower the activation threshold of autoreactive B cells. In this setting, PF4–polyanion complexes act as a self-antigen presented in an immunogenic context, favouring clonal selection rather than deletion or anergy. Although direct evidence for checkpoint failure in humans is limited, this framework aligns VITT with established principles of autoimmunity and supports its use as a human model to study vaccine-triggered immune dysregulation.

### 4.4. Fc-Mediated Platelet Activation and Thrombo-Inflammation

Once generated, anti-PF4 IgG antibodies form immune complexes that bind to FcγRIIa receptors on platelets, triggering powerful activation signals [3,5,14]. This leads to platelet aggregation, degranulation, surface expression of procoagulant phosphatidylserine, and the release of platelet-derived extracellular vesicles rich in tissue factor and negatively charged phospholipids [14,24]. These changes markedly enhance thrombin generation and fibrin formation, driving widespread thrombosis.

In parallel, immune complexes activate the complement cascade, further amplifying platelet activation and endothelial injury [27]. Neutrophils are recruited and stimulated through Fc and complement receptors, leading to the release of neutrophil extracellular traps (NETs), which provide a scaffold for thrombus formation and propagate inflammation [28]. Endothelial cells exposed to these inflammatory mediators upregulate adhesion molecules and tissue factor, perpetuating a feed-forward loop of immune-thrombosis [17,18].

These interconnected mechanisms are described in detail throughout the text to allow a fully self-contained understanding of the VITT immunopathogenic cascade.

### 4.5. A Self-Amplifying Pathogenic Loop

The convergence of platelet activation, complement signaling, NET formation, and endothelial dysfunction establishes a self-sustaining thrombo-inflammatory circuit that explains the rapid clinical deterioration observed in VITT [15,16,17,18]. Importantly, this cascade is largely independent of classical coagulation triggers and instead reflects a primary immune-driven mechanism, positioning VITT as a prototypical model of antibody-mediated immuno-thrombosis.

Collectively, these mechanisms illustrate how a protective vaccine-triggered immune response can, in rare individuals, be redirected toward a catastrophic autoimmune process. VITT therefore represents not only a novel adverse event but also a powerful model for studying the intersection between innate immunity, autoimmunity, and thrombosis [6,7,10].

**Figure 1:**
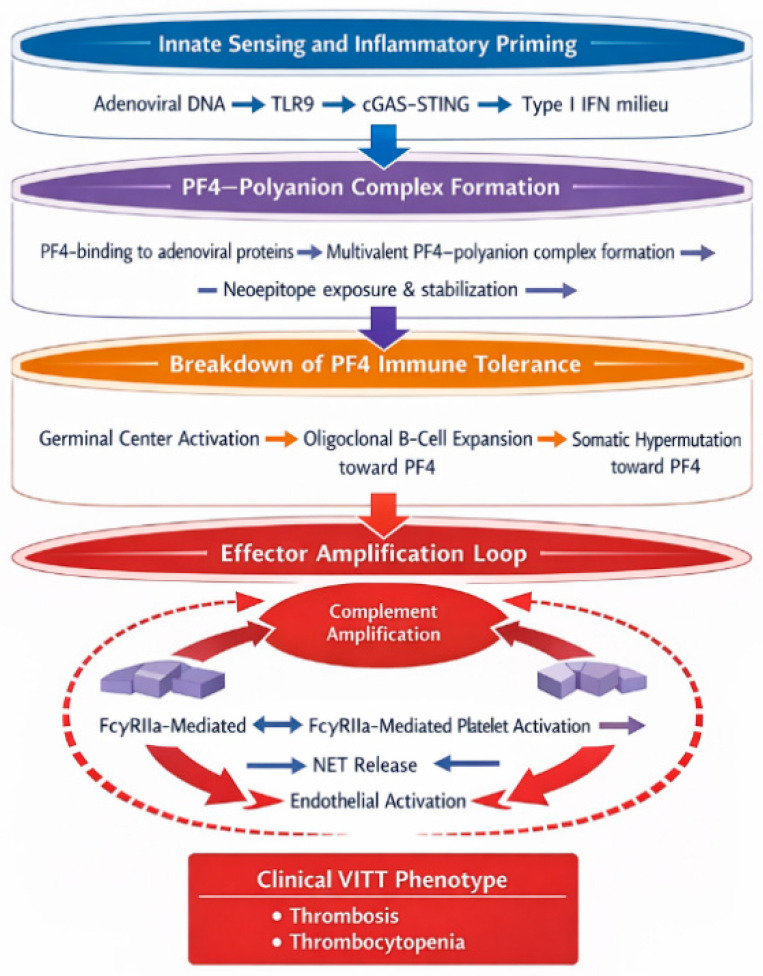
Multilevel immunopathogenic network model of vaccine-induced immune thrombotic thrombocytopenia (VITT). Legend. VITT is represented as a multilayered immune network rather than a linear cascade. Adenoviral DNA sensing (TLR9, cGAS–STING) promotes inflammatory priming and facilitates PF4–polyanion complex formation. Subsequent breakdown of PF4 immune tolerance leads to high-affinity anti-PF4 IgG production. Downstream FcγRIIa-mediated platelet activation, complement amplification, NET release, and endothelial activation form a self-reinforcing effector loop culminating in thrombosis and thrombocytopenia. Dominant pathways are visually emphasized to reflect probabilistic weighting within the network.

To facilitate integration of the multistep immunopathogenic cascade described above, Figure 1 provides a schematic representation of the core VITT model, illustrating PF4–polyanion complex formation, adaptive anti-PF4 IgG generation, FcγRIIa-mediated platelet activation, complement amplification, NET release, endothelial perturbation, and the establishment of a self-amplifying thrombo-inflammatory loop. Unlike earlier schematics that depicted a linear cascade, Figure 1 emphasizes bidirectional amplification loops and the integration of innate sensing pathways (TLR9, cGAS–STING) upstream of adaptive tolerance breakdown. This representation intentionally shifts the model from a static antibody-driven event to a systems-level immune network in which antigen structure, innate activation, and Fc-mediated effector pathways converge.

## 5. VITT in the Context of HIT and COVID-19-Associated Coagulopathy

Vaccine-induced immune thrombotic thrombocytopenia (VITT) shares important mechanistic features with other immune-mediated thrombotic disorders, particularly heparin-induced thrombocytopenia (HIT), while also displaying critical differences from the coagulopathy observed in severe COVID-19 [8,9,30,31]. Comparative analysis of these conditions provides valuable insight into how distinct immune triggers can converge on common thrombo-inflammatory pathways.

Rather than relying on schematic representation, the following comparison emphasizes conceptual and mechanistic differences directly within the narrative framework. A structured comparison of triggers, immune effectors, and clinical patterns across VITT, HIT, and COVID-19-associated coagulopathy is provided in Table 2.

### 5.1. Comparison with Heparin-Induced Thrombocytopenia

HIT is a well-characterized, antibody-mediated prothrombotic disorder that arises following exposure to heparin. In HIT, PF4-heparin complexes act as neoantigens, inducing IgG antibodies that activate platelets via FcγRIIa, leading to thrombocytopenia and thrombosis [8]. VITT mirrors this fundamental mechanism, as both conditions involve pathogenic anti-PF4 antibodies that trigger platelet activation and thrombin generation in the absence of classical coagulation defects [6,9].

Despite these similarities, several key differences distinguish VITT from HIT. First, the triggering antigen in VITT is not heparin but vaccine-derived polyanionic constituents, including adenoviral proteins and DNA [11,12,13]. Second, anti-PF4 antibodies in VITT display broader epitope recognition and higher binding avidity compared with those observed in HIT, which may explain the unusually severe clinical phenotype [19,30]. Third, VITT antibodies often strongly activate platelets even in the absence of heparin, whereas HIT antibodies typically require heparin or PF4-heparin complexes for optimal activation [8,30].

Clinically, VITT is associated with a higher frequency of atypical thrombotic sites, particularly cerebral venous sinus and splanchnic vein thrombosis, and with more profound thrombocytopenia and consumptive coagulopathy [1,4]. These features suggest a more aggressive immune activation and systemic inflammatory response in VITT than in classical HIT.

Beyond shared molecular pathways, VITT and heparin-induced thrombocytopenia differ in their broader immunological framing. HIT represents a drug-dependent autoimmune reaction with a well-defined exogenous trigger and predictable risk factors, whereas VITT arises in the context of vaccine-induced immune activation without a prior history of antigen exposure. This distinction has important implications for understanding immune priming, tolerance, and reversibility.

In HIT, discontinuation of the triggering agent and immune modulation typically lead to antibody waning and clinical resolution. In contrast, the dynamics of anti-PF4 antibodies in VITT appear more heterogeneous, with emerging evidence of prolonged seropositivity in some patients. These differences suggest that, despite mechanistic overlap, VITT may represent a more complex and context-dependent form of immune dysregulation.

### 5.2. Distinction from COVID-19-Associated Coagulopathy

Severe COVID-19 is frequently complicated by a hypercoagulable state characterized by elevated D-dimer levels, microvascular thrombosis, and platelet hyperreactivity [7,31]. The cited COVID-19 studies are referenced here to illustrate shared pathways of immune-thrombosis (e.g., platelet activation, NET formation, complement engagement), rather than to imply direct mechanistic equivalence with VITT. The comparison serves a conceptual purpose, highlighting convergence at the level of effector pathways despite distinct initiating triggers. However, the underlying pathophysiology of COVID-19-associated coagulopathy differs fundamentally from that of VITT. In COVID-19, thrombosis is primarily driven by endothelial injury, cytokine storm, and dysregulated innate immune responses rather than by a specific autoantibody-mediated mechanism [7,31].

Although platelet activation and extracellular vesicle release are common to both conditions, COVID-19-associated coagulopathy lacks the defining anti-PF4 antibodies and the heparin-independent platelet activation that characterize VITT [7,9]. Furthermore, thrombocytopenia in COVID-19 is typically mild to moderate and reflects consumptive and inflammatory mechanisms, whereas in VITT it is a direct consequence of antibody-mediated platelet clearance and activation [3,5].

The comparison with COVID-19-associated coagulopathy further underscores the conceptual uniqueness of VITT. While both conditions involve immuno-thrombosis, endothelial perturbation, and innate immune activation, the presence of a highly specific autoantibody response in VITT establishes a direct causal link between adaptive immunity and thrombosis. In contrast, COVID-19-associated coagulopathy reflects a multifactorial process driven by viral infection, systemic inflammation, and endothelial injury, without a single dominant autoimmune trigger.

This distinction highlights why therapeutic strategies and prognostic considerations cannot be directly extrapolated between the two conditions and reinforces the value of VITT as a model system for studying antibody-mediated immuno-thrombosis.

### 5.3. Shared and Divergent Pathways

Together, HIT, VITT, and COVID-19-associated coagulopathy illustrate how diverse immune and inflammatory triggers can converge on common thrombo-inflammatory pathways involving platelets, neutrophils, complement, and the endothelium [12,17,18]. However, VITT is unique in that it represents a vaccine-triggered autoimmune disorder, in which a self-protein becomes the primary antigenic target, driving a fulminant, antibody-dependent immune-thrombotic cascade [6,10].

This comparison underscores the conceptual value of VITT as a model of immune-mediated thrombosis, bridging the fields of autoimmunity, vaccinology, and hemostasis.

Taken together, the comparison between VITT, HIT, and COVID-19-associated coagulopathy illustrates how distinct immune triggers can converge on partially overlapping thrombo-inflammatory pathways while retaining fundamentally different immunological architectures. Among these entities, VITT is unique in that it represents a vaccine-triggered, antibody-mediated autoimmune syndrome with a defined antigenic target and effector mechanism. This positioning reinforces its value not only as a clinical entity but also as a conceptual bridge linking autoimmunity, vaccinology, and immuno-thrombosis.

## 6. Why Is VITT Platform-Enriched in Adenoviral Vector Vaccines?

One of the most intriguing aspects of VITT is its strong epidemiological enrichment within adenoviral vector platforms. However, given the rarity of the syndrome and differential surveillance intensity across vaccine types and jurisdictions, current evidence supports probabilistic enrichment rather than definitive qualitative exclusivity [32].

Although current epidemiological and mechanistic evidence strongly supports a platform-enriched association of classic VITT with adenoviral vector vaccines, it is important to acknowledge the limitations inherent to studying an extremely rare adverse event. Statistical power is limited, and diagnostic, reporting, and publication biases may influence apparent platform specificity. In addition, thrombotic events and PF4-reactive antibodies have been reported after non-adenoviral vaccine platforms, even if they do not fulfil the full VITT phenotype. Recognizing this mechanistic “border zone” is essential to avoid overly closed or non-falsifiable models and to clearly distinguish dominant pathways from rarer or atypical presentations.

This observation suggests that specific properties of adenoviral vectors, rather than the spike protein antigen itself, are critical for initiating the immunopathogenic cascade leading to VITT.

Adenoviral vectors possess unique structural and immunological features that distinguish them from lipid nanoparticle-encapsulated mRNA vaccines. First, adenoviral capsids and their associated nucleic acids carry strong negative charges, enabling them to function as polyanions capable of binding PF4 with high affinity [11,12,13]. In vitro studies have demonstrated that adenoviral particles readily form stable multimolecular complexes with PF4, whereas lipid nanoparticles and mRNA alone do not show comparable binding properties [12]. These PF4-adenovirus complexes expose cryptic epitopes that can be recognized by anti-PF4 antibodies, thereby providing the initial trigger for immune sensitization [6,10].

Second, adenoviral vectors display a broader systemic biodistribution following intramuscular injection compared with mRNA vaccines, which are largely retained at the injection site and the draining lymph nodes [33]. Low-level systemic dissemination of adenoviral particles may increase the likelihood of direct interactions with circulating platelets and PF4, facilitating the formation of immunogenic complexes in the bloodstream [11,34].

Third, adenoviral DNA acts as a potent activator of innate immune sensors, particularly Toll-like receptor 9 (TLR9) and the cytosolic cGAS–STING pathway, which drive robust type I interferon responses and pro-inflammatory cytokine production [35]. This strong innate immune activation provides an adjuvant-like effect that lowers the threshold for breaking immune tolerance to self-antigens such as PF4 [6,10]. In contrast, mRNA vaccines are designed to minimize innate immune activation through nucleoside modification and optimized lipid formulations, thereby reducing the risk of excessive inflammatory signaling [32].

While the association between adenoviral vectors and classic VITT is compelling, a strictly causal interpretation based solely on residual DNA or polyanionic content may be overly simplistic. Sporadic reports of PF4-reactive antibodies and thrombotic events following mRNA-based vaccines, although not meeting full VITT diagnostic criteria, indicate that PF4 immunogenicity is not exclusively confined to adenoviral platforms.

Additional variables, including antigen dose, biodistribution, and route of administration, may modulate immune exposure to PF4-containing complexes and influence risk at the extremes. Intramuscular delivery, local vascular leakage, and inadvertent intravascular injection have all been proposed as modifiers of systemic immune activation, although direct human data remain limited. Acknowledging these factors reinforces a probabilistic, rather than absolute, model of platform specificity and highlights the need for mechanistic studies that integrate formulation, delivery, and host factors. Finally, manufacturing-related factors, including residual host–cell proteins, free DNA, and other polyanionic impurities, may further contribute to the immunogenicity of PF4-containing complexes in adenoviral vaccines [13]. Although present at extremely low levels, these components may synergize with adenoviral particles to amplify immune recognition in susceptible individuals.

Collectively, these features provide a plausible mechanistic explanation for the observed platform-enriched association of classic VITT with adenoviral vector vaccines, while leaving open important questions regarding atypical phenotypes, rare exceptions, and host-dependent modifiers of risk. They also highlight the importance of understanding how vaccine components interact with endogenous proteins to shape immune responses. Insights gained from VITT may therefore inform the rational design of future vaccine platforms with improved safety profiles, minimizing the risk of rare but severe immune-mediated adverse events [10].

**Figure 2:**
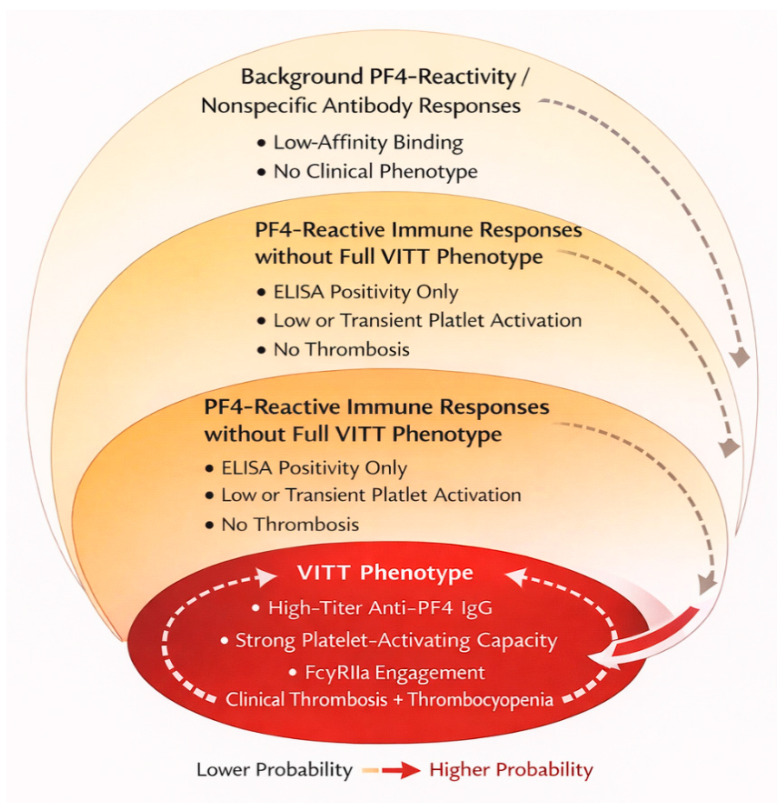
Probabilistic spectrum model of PF4-reactive immune responses following adenoviral vector vaccination. Legend. This schematic illustrates VITT as the highest-probability expression within a spectrum of PF4-reactive immune responses. Peripheral zones represent low-affinity or transient antibody responses without clinical manifestations, whereas intermediate zones depict ELISA positivity without sustained platelet-activating capacity. The central domain corresponds to high-titer, platelet-activating anti-PF4 IgG associated with thrombosis and thrombocytopenia. The graded structure reflects probabilistic enrichment rather than qualitative exclusivity.

Figure 2 schematically summarizes the current probabilistic model of platform enrichment, illustrating the mechanistic factors favoring PF4 immunogenicity in adenoviral vector vaccines (polyanionic capsid/DNA interactions, systemic biodistribution, innate immune activation), while also depicting the proposed “border zone” in which rare or incomplete PF4-reactive phenotypes may occur outside the classic VITT framework. Importantly, the model distinguishes dominant mechanistic pathways (PF4–adenoviral complex formation and FcγRIIa-mediated activation) from peripheral or low-probability pathways represented within the shaded “border zone.” This layered visualization reflects a probabilistic hierarchy rather than a binary classification and underscores that qualitative exclusivity remains unproven.

In the revised graphic, dominant pathways are visually weighted (thicker arrows/central nodes), whereas peripheral mechanisms are intentionally de-emphasized within the shaded border zone to reflect their lower-probability contribution.

However, the current evidence base has important limitations that temper definitive conclusions regarding platform specificity. First, the rarity of VITT inherently limits statistical power, and absence of evidence cannot be equated with evidence of absence. Second, differential surveillance intensity across vaccine platforms and countries may have influenced detection rates. Third, mechanistic studies demonstrating PF4 binding to adenoviral particles are largely based on in vitro systems, and their quantitative relevance in vivo remains incompletely established. Future studies should integrate standardized functional assays, comparative biodistribution analyses, and immunogenetic profiling across vaccine platforms. Only through such multidimensional approaches will it be possible to determine whether adenoviral vectors are uniquely capable of breaking tolerance to PF4 or whether they represent a quantitatively enriched, but not qualitatively exclusive, trigger.

Definitive confirmation of qualitative exclusivity would require (a) standardized functional anti-PF4 testing across platforms, (b) harmonized pharmacovigilance with uniform case definitions, and (c) mechanistic demonstration that non-adenoviral formulations cannot generate structurally comparable PF4–polyanion complexes in vivo. At present, such evidence remains incomplete.

## 7. Clinical Implications and Therapeutic Strategies

Beyond its mechanistic interest, VITT has had immediate and lasting implications for clinical practice, particularly in the acute management of thrombosis accompanied by thrombocytopenia. The rapid translation of immunopathogenic insights into diagnostic algorithms and therapeutic strategies represents a notable success of bench-to-bedside medicine during the COVID-19 pandemic. At the same time, the rarity of the condition and the absence of randomized controlled trials necessitate cautious interpretation of available evidence and emphasize the role of expert consensus and real-world data in guiding care.

The recognition of vaccine-induced immune thrombotic thrombocytopenia (VITT) has had profound implications for clinical practice, vaccine safety surveillance, and the management of immune-mediated thrombotic disorders. A pragmatic stepwise approach is summarized in Table 3. Early in the pandemic, the high mortality associated with VITT underscored the need for rapid diagnostic pathways and evidence-based treatment strategies [1,14,20,23]. The hallmark features of thrombocytopenia, markedly elevated D-dimer levels, and anti-PF4 antibodies detected by enzyme-linked immunosorbent assay (ELISA) now form the basis of diagnostic algorithms used worldwide [4,18].

Prompt initiation of therapy is critical. High-dose intravenous immunoglobulin (IVIG) represents the cornerstone of treatment, as it competitively inhibits FcγRIIa on platelets, thereby blocking immune complex-mediated platelet activation [14,24]. Non-heparin anticoagulation, including direct oral anticoagulants, fondaparinux, or argatroban, is recommended to prevent thrombus propagation while avoiding heparin, which may exacerbate antibody-mediated platelet activation [14]. In severe or refractory cases, adjunctive therapies such as corticosteroids, plasma exchange, or complement inhibition have been employed, although robust randomized data are lacking [34].

Beyond the acute phase, recent prospective and cohort follow-up studies have provided clinically actionable data for long-term management. Most survivors appear to have a low risk of recurrent thrombosis or thrombocytopenia, although a subset may show prolonged laboratory persistence of anti-PF4 antibodies, including platelet-activating reactivity, supporting individualized duration of anticoagulation and follow-up testing [36,37]. Reassuringly, subsequent vaccination with mRNA-based vaccines has not been associated with clinical relapse in reported cohorts and case series, suggesting that continued immunization can be considered with appropriate specialist supervision [36].

### 7.1. Follow-Up, Duration of Anticoagulation, and Re-Exposure Considerations

Beyond acute management, several practical questions remain debated and are increasingly relevant to contemporary clinical care. First, the optimal duration of anticoagulation after VITT is not fully standardised and is often individualised based on thrombotic burden, platelet recovery, D-dimer normalisation, and the evolving understanding of anti-PF4 antibody persistence. Second, follow-up strategies vary across centres with respect to repeat anti-PF4 testing and functional assays, reflecting uncertainty about the relationship between persistent seropositivity and ongoing pathogenic potential [38]. Re-vaccination after VITT represents another clinically important area in which evidence remains limited. In practice, subsequent vaccination decisions often consider platform selection, risk–benefit context, and patient-specific factors, but robust predictors of recurrence are lacking. These uncertainties highlight the need for harmonised follow-up protocols and prospective data collection to define long-term risk and to support evidence-based guidance [38].

Beyond acute management, VITT has reshaped regulatory and pharmacovigilance frameworks. Active post-marketing surveillance systems and rapid international data sharing enabled the timely identification of this rare adverse event, guiding vaccine policy decisions and risk stratification strategies [10,12]. Importantly, the benefits of COVID-19 vaccination overwhelmingly outweigh the risks of VITT, and adenoviral vaccines remain valuable tools in many global settings [28].

From a translational perspective, VITT has illuminated fundamental principles of immune-mediated thrombosis, emphasizing the interplay between innate immune activation, autoantibody generation, and platelet-driven thrombo-inflammation [6,7]. These insights extend to other autoimmune and inflammatory disorders and may inform the development of targeted therapies that modulate Fc receptor signaling, complement activation, or NET formation [17,18].

Management during pregnancy represents a particular challenge, given limited data and the need to balance maternal thrombotic risk with fetal safety. In such settings, non-heparin anticoagulants with established pregnancy safety profiles are generally preferred, while multidisciplinary management is essential.

With respect to anticoagulant choice, both direct oral anticoagulants (DOACs) and parenteral direct thrombin inhibitors have been used successfully. DOACs offer practical advantages for long-term treatment, whereas parenteral agents may be preferred in unstable or critically ill patients. Comparative data remain limited, and treatment decisions should be individualized.

While early initiation of non-heparin anticoagulation and high-dose intravenous immunoglobulin has become standard of care, management beyond the acute phase remains less clearly defined.

Follow-up strategies also vary widely. As discussed in Section Long-Term Evolution and Post-Policy Landscape (2023–2025), longitudinal studies indicate that ELISA-detectable anti-PF4 IgG may persist despite loss of platelet-activating capacity. In the clinical setting, this distinction has practical implications: anticoagulation duration and follow-up strategies should not rely on serological persistence alone but on integrated clinical and functional assessment.

Accordingly, where available, functional platelet-activation testing can be used selectively to support follow-up decisions in clinically ambiguous scenarios rather than as routine serial monitoring.

### 7.2. Long-Term Management, Antibody Persistence, and Re-Vaccination

A central biological question emerging from long-term follow-up concerns the immunological behavior of anti-PF4 antibodies after clinical recovery. Beyond clinical management considerations discussed above, the persistence, clonal evolution, and functional capacity of these antibodies provide insight into the stability or reversibility of the autoimmune response triggered during acute VITT. The long-term immunological behavior of anti-PF4 antibodies has been detailed above (Section Long-Term Evolution and Post-Policy Landscape (2023–2025)). From a practical standpoint, current data suggest that persistence of binding antibodies does not necessarily imply sustained pathogenic autoimmunity.

The key unmet need is a validated, prospective risk-stratification approach combining clinical phenotype, imaging burden, and selected laboratory markers (including functional assays where available). Until such data emerge, follow-up and treatment duration should remain phenotype-driven and guideline-consistent.

Re-vaccination after VITT represents another clinically important and unresolved issue. Available experience suggests that re-exposure to non-adenoviral platforms may be safe in selected cases, but robust predictors of recurrence are absent, and systematic data are limited. These gaps highlight the need for harmonized follow-up protocols and prospective registries to inform long-term risk stratification and vaccination strategies in patients with prior VITT.

Management of VITT in pregnancy or in individuals of childbearing potential poses additional challenges, given the paucity of data and the need to balance maternal thrombotic risk with fetal safety. In such scenarios, multidisciplinary management involving hematology, obstetrics, and maternal-fetal medicine is essential. Non-heparin anticoagulants with established pregnancy safety profiles are generally preferred, while treatment decisions must be individualized in the absence of high-quality comparative evidence.

Similar considerations apply to other special populations, including patients with severe renal impairment or those requiring invasive procedures. These settings further highlight the importance of flexible, mechanism-informed treatment strategies rather than rigid algorithms.

## 8. Future Research Directions

Despite rapid advances in understanding VITT, several priorities can now be stratified into short-term clinically actionable goals and longer-term mechanistic investigations.

### 8.1. Short-Term Priorities (Clinically Actionable)

These include (a) standardization of follow-up protocols integrating clinical evolution with functional platelet activation assays rather than ELISA alone; (b) prospective registries to define optimal duration of anticoagulation; (c) harmonized criteria for safe re-vaccination; and (d) refinement of laboratory algorithms capable of distinguishing pathogenic from non-pathogenic anti-PF4 responses. Addressing these issues is directly relevant to patient safety, regulatory decision-making, and evidence-based guidance.

### 8.2. Intermediate Translational Priorities

These involve defining biomarkers of susceptibility, including HLA associations, B-cell repertoire signatures, and markers of innate immune activation, as well as comparative studies of vaccine formulation, dose, and biodistribution.

### 8.3. Long-Term Mechanistic Priorities

At a deeper level, VITT provides a unique human model to explore how multivalent antigen presentation, innate immune sensing, and tolerance checkpoints intersect to generate autoimmunity. Elucidating the structural determinants of PF4 immunogenicity, the role of germinal center dynamics, and the reversibility of autoreactive B-cell clones may generate insights extending beyond vaccinology into broader autoimmune pathophysiology.

By explicitly separating urgent clinical questions from longer-term mechanistic exploration, this framework clarifies which uncertainties are immediately relevant for vaccine safety and which represent broader immunological frontiers.

## 9. Conclusions

Vaccine-induced immune thrombotic thrombocytopenia represents a rare but highly informative model of dysregulated vaccine-triggered immunity. Through the formation of immunogenic PF4-polyanion complexes and the generation of pathogenic anti-PF4 antibodies, a protective immune response is redirected toward a fulminant autoimmune thrombo-inflammatory cascade. The resulting platelet, complement, neutrophil, and endothelial activation explains the paradoxical coexistence of thrombosis and thrombocytopenia that defines this syndrome.

Beyond its clinical relevance, VITT provides a conceptual framework for understanding how innate immune sensing, adjuvant effects, and molecular mimicry can converge to break immune tolerance. The platform-enriched association of this syndrome to adenoviral vector platforms highlights the importance of vaccine composition, biodistribution, and innate immune activation in shaping immunogenicity and safety.

Revisiting VITT in 2025 is valuable not merely for updated epidemiology, but for testing the stability of earlier models against longitudinal and mechanistic evidence. The field has moved from binary interpretations (ELISA positivity = pathogenicity; adenoviral vectors = exclusivity) toward a probabilistic framework that separates binding from function and enrichment from exclusivity. Future progress will depend on harmonized prospective cohorts, standardized functional phenotyping, and integrated immunogenetic studies to define susceptibility and long-term immune regulation.

## Figures and Tables

**Table 1:** Key clinical, laboratory, and immunological features of vaccine-induced immune thrombotic thrombocytopenia.

Feature	Typical Findings in VITT
Time from vaccination	5–30 days (median 10–14)
Platelet count	<50 × 10^3^/μL
D-dimer	Markedly elevated (>20,000 ng/mL)
Fibrinogen	Reduced
Anti-PF4 IgG	High-titer, heparin-independent
Thrombosis sites	CVST, splanchnic veins, pulmonary arteries
Mortality (early reports)	>40%
Mortality (recent cohorts)	<10–15%

Legend: Summary of the main clinical and laboratory features characterizing VITT, reflecting the coexistence of thrombosis and consumptive thrombocytopenia driven by anti-PF4 immune complexes.

**Table 2:** Mechanistic differences and similarities between VITT, HIT, and COVID-19-associated coagulopathy.

Feature	VITT	HIT	COVID-19 Coagulopathy
Trigger	Adenoviral 19.	Heparin	SARS-CoV-2 infection
Autoantibodies	Anti-PF4 IgG	Anti-PF4/heparin IgG	Absent
Platelet activation	FcγRIIa-mediated	FcγRIIa-mediated	Inflammatory
Complement	Strongly activated	Activated	Activated
NETs	Prominent	Present	Prominent
Endothelial injury	Immune-mediated	Secondary	Direct viral/cytokine
Thrombosis sites	Atypical	Venous/arterial	Microvascular/venous

Legend: Comparison of the main immune-thrombotic pathways involved in VITT, HIT, and COVID-19-associated coagulopathy, highlighting both shared mechanisms and disease-specific features.

**Table 3:** Practical approach to initial and early management of vaccine-induced immune thrombotic thrombocytopenia (VITT).

Step	Recommendation
Suspected VITT	Check platelet count, D-dimer, fibrinogen, anti-PF4 ELISA
Anticoagulation	Start non-heparin anticoagulation (DTI or DOAC)
Immune modulation	High-dose IVIG (1 g/kg for 2 days)
Severe/refractory cases	Consider steroids, plasma exchange
Follow-up	Individualised anticoagulation duration, clinical and laboratory monitoring

Legend: This table summarises a pragmatic, stepwise approach to the diagnosis and management of suspected and confirmed VITT, integrating laboratory assessment, early anticoagulation, immune-modulating therapy, and follow-up considerations. Recommendations reflect current expert consensus and observational evidence rather than randomized controlled trials and should be individualised according to clinical severity, comorbidities, and local resource availability. DTI: Direct Thrombin Inhibitor.

## Data Availability

No new data were created or analyzed in this study.
